# Progress in tuberculosis diagnosis and laboratory services in the Western Pacific Region: a situational analysis of seven high tuberculosis burden countries

**DOI:** 10.1186/s41182-025-00898-z

**Published:** 2026-05-18

**Authors:** Emily Lai-Ho MacLean, Kyung Hyun Oh, Kalpeshsinh Rahevar, Carl-Michael Nathanson, Alexei Korobitsyn, Satoshi Mitarai, Seiya Kato, Fukushi Morishita, Huong Thi Gian Tran, Rajendra Prasad Hubraj Yadav

**Affiliations:** 1 NHMRC Clinical Trials Centre, Faculty of Medicine and Health, University of Sydney, Sydney, Australia; 2 WHO Collaborating Centre for Tuberculosis, Sydney Infectious Diseases Institute, University of Sydney, Sydney, Australia; 3 World Health Organization Regional Office for the Western Pacific, Manilla, Philippines; 4 Global Programme on Tuberculosis & Lung Health, World Health Organization, Geneva, Switzerland; 5 The Research Institute of Tuberculosis, Japan Anti-Tuberculosis Association, Tokyo, Japan

**Keywords:** Tuberculosis, Western Pacific region, Diagnostics, Laboratory services, National tuberculosis programmes

## Abstract

**Background:**

Timely, accurate tuberculosis (TB) diagnostics and strong laboratory networks are critical for providing high-quality TB care. In the Western Pacific Region, available resources, TB burdens, and access to TB testing vary dramatically between countries. To understand the current regional situation, we described TB diagnostic test availability and laboratory services in seven countries with high TB burdens in the Western Pacific Region:

**Main text:**

In 2024, World Health Organization’s (WHO) Western Pacific Regional Office conducted an assessment of TB diagnostic test availability and laboratory services in seven countries with high TB burdens: Cambodia, China, Lao People’s Democratic Republic, Mongolia, Philippines, Papua New Guinea, and Viet Nam. Standardised surveys were sent to members of country national tuberculosis programmes, and follow-up interviews were conducted. An in-person workshop was attended where preliminary findings were presented and updated, if necessary. The exercise revealed a high uptake of WHO-endorsed rapid molecular tests for TB detection, although the use of smear microscopy persists in most countries’ remote areas. Regarding drug susceptibility testing, both molecular and phenotypic methods are employed. Testing for first-line TB drug resistance is generally available, but currently, testing capacity for new and re-purposed drugs remains limited. Most countries provide TB testing at district-level facilities in conjunction with well-established specimen transport networks, with laboratories utilising a mix of paper and electronic databases. Comprehensive TB and drug-resistance testing at peripheral settings remains a rarity.

**Conclusions:**

The Western Pacific Region has invested significantly in TB testing and laboratory services. To ensure everyone in the region has access to TB diagnostics, these efforts must be maintained.

**Supplementary Information:**

The online version contains supplementary material available at 10.1186/s41182-025-00898-z.

## Background: tuberculosis testing and cases in the region

Globally, tuberculosis (TB) is one of the most serious public health challenges and has resurged as the top infectious disease killer [1]. Robust diagnostic and laboratory services are essential for countries to find and adequately manage people with TB [2]. At the same time, these systems must be accessible and readily available for individuals to receive timely and accurate diagnoses so they may commence appropriate, effective treatment [3]. Progress towards ensuring universal access to high-quality TB diagnostics and laboratory services has been uneven between countries. Challenges such as limited country-based expertise and qualified workforce, inadequate infrastructure, and outdated data management approaches persist. To compound these existing matters, the coronavirus disease 2019 (COVID-19) pandemic diverted significant resources from other disease programmes in 2020–2023 [4]. The negative impact of the pandemic on TB disease control continues to resonate worldwide even today [5].

Year-on-year epidemiological data [6, 7] and previous work investigating TB services [8] indicate that the World Health Organization (WHO) Western Pacific Region is progressing in expanding the reach of TB services. However, the regional epidemiological situation remains difficult. During the COVID-19 pandemic, there was a marked decline in TB case notifications across the Western Pacific Region in 2020 and 2021 [5, 9]. In response, national TB programmes (NTPs) have worked to address this issue and begin to recover [6, 9]. Many countries have invested substantially in diagnostic capacity and laboratory services [10, 11] resulting in increasing TB case detection. In 2023, TB incidence increased in the Region, as 1.39 million newly diagnosed TB patients were notified, the same level as in 2019 [5].

Between 2015 and 2020, the estimated TB-related mortality declined 13% from 5.3 to 4.5 per 100,000 population [7], before sharply increasing to 5 per 100,000 population in 2021 [12]. In 2023, the rate has returned to 2020 levels [13]. These large and rapid fluctuations in mortality indicate that accessing TB services, including diagnosis and treatment, was extremely challenging during the pandemic. The lasting impact and potential changes to services across the entire region have not been examined, especially in the context of resources being increasingly channelled away from TB programs to other public health areas.

Considering the changing landscape, there is a need to understand the availability of TB diagnostics to better inform TB control efforts in the Western Pacific Region. A regional assessment of the implementation of TB diagnosis and laboratory services in the Western Pacific Region was commenced in January 2024 to provide current insight into TB diagnostics and laboratory systems and identify gaps and challenges. The assessment aimed to inform prioritisation and resource allocation, enabling countries to reduce TB incidence and mortality and ultimately reach the goals described in the Western Pacific Regional Framework to End TB: 2021–2030 [14].

## Main text

### Data collection and consultation

A survey was conducted to understand the status of TB laboratory and diagnostic services in seven high-priority countries in the Western Pacific Region. Five of the selected countries were China, Mongolia, Papua New Guinea, the Philippines, and Viet Nam, which are all included in the 2021–2025 WHO list of high-burden countries for TB in the Western Pacific Region [15]. The other two included countries were Cambodia and Lao People’s Democratic Republic (Lao PDR), both of which have large total numbers of TB cases.

Briefly, a structured survey was developed based on key indicators of interest identified by the WHO Western Pacific Regional Office (WPRO). The survey had three sections: (i) TB test availability and usage; (ii) access to TB diagnostics; and (iii) laboratory systems. Parts (i) and (iii) of the survey were created and shared through a cloud-based data collection system, KoboToolbox [16]. A formatted and editable Excel sheet was used for part (ii). Questions in parts (i) and (iii) were primarily multiple-choice selections with space for explanatory text. Part (ii) comprised the 12 benchmarks defined in the 2023 WHO Standard: Universal Access to Rapid Tuberculosis Diagnostics [17]. The draft survey was shared with members of WPRO and the WHO headquarters for further feedback and refining. Once final, a representative from the WHO WPRO distributed the survey via email to staff of WHO country offices, members of country NTPs, and national TB reference laboratories in the seven countries. It was open from 8 February 2024 until 15 March 2024.

After receipt of survey responses, interviews were conducted with staff from the WHO country office and technical focal points from NTPs or their delegates. Follow-up questions were posed to update any discrepant survey responses, gain further country context and explanation of survey answers, and clarify the countries’ TB diagnostic landscape, test access, and laboratory systems.

The results were presented at the WHO Regional Workshop on TB Diagnosis and Laboratory Services in the Western Pacific Region, held at Japan Anti-Tuberculosis Association in Tokyo, Japan, on 16 to 18 April 2024. The workshop was attended by staff from NTPs and local WPRO offices of the seven high-priority countries, members from WHO headquarters, and observers from Japan Research Institute of Tuberculosis. At this workshop, countries had the opportunity to comment further on the survey findings and correct any inaccuracies.

### TB diagnostics usage and availability

The availability of the various WHO-defined classes of TB diagnostic tests and drug susceptibility tests [18] within seven countries’ NTPs is summarised in Table 1. Note that only tests that were reportedly available in at least one country are included in Table 1 (i.e. the class of moderate complexity automated nucleic acid amplification tests for the detection of TB and resistance to rifampicin and isoniazid [18] was not available in any country and therefore is not included in Table 1).

**Table 1:**
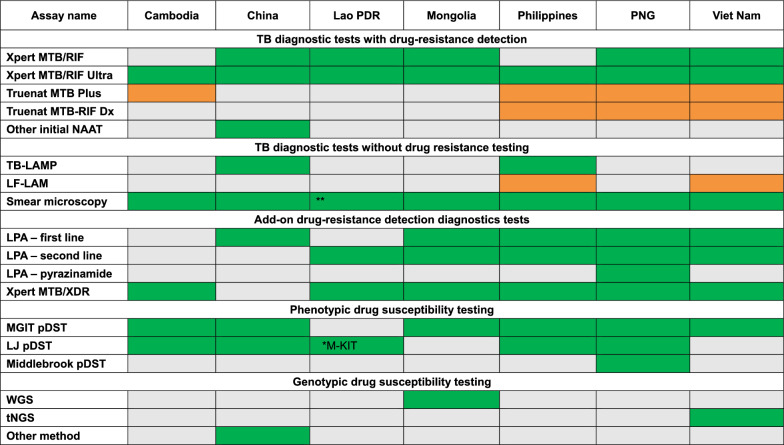
TB tests available in seven high-priority countries in the Western Pacific Region for TB diagnosis and drug-resistance detection

*Green* assay is available through programmatic implementation, *orange* assay is available on project-based or for research use only, *grey* assay is unavailable

*LF-LAM* lateral flow lipoarabinomannan, *LJ* Löwenstein–Jensen medium, *LPA* line probe assay, *NAAT* nucleic acid amplification test, *pDST* phenotypic drug susceptibility testing, *PNG* Papua New Guinea, *tNGS* targeted next generation sequencing, *WGS* whole genome sequencing

**Available for treatment monitoring only

For initial diagnostic tests for TB with drug-resistance detection, all countries reported programmatic implementation of Xpert MTB/RIF and/or Xpert MTB/RIF Ultra ([Xpert] Cepheid, USA) (Table 1). NTP guidelines include Xpert as an initial test for TB detection for everyone with presumptive TB in all countries (Table 2), except in Viet Nam. There, the NTP requires that adults at low risk for TB first undergo chest radiography before Xpert testing. This means all countries are providing some up-front drug-resistance testing, as Xpert can also detect resistance to rifampicin. In Cambodia, the Philippines, Papua New Guinea, and Viet Nam, Truenat MTB Plus (Truenat, Molbio, India) is also available (Table 1), typically through donors (i.e. USAID in Cambodia, the Philippines, and Viet Nam) or through research activities with partners (i.e. Burnett Institute in Papua New Guinea). Xpert and Truenat generally are available to the same population (Table 2), so the choice of whether to use Xpert or Truenat is primarily a pragmatic or logistical one; for example, the decision may be based on expiration dates of available kits. Only China reported using additional TB diagnostic tests with drug-resistance detection as initial diagnostic tests. Over 30 Chinese Food and Drug Administration (CFDA)-endorsed nucleic acid amplification tests (NAATs) are being used throughout the country, such as cross-primer amplification assays and real-time PCR-based NAATs (Table 1).

**Table 2:** Populations eligible for testing on TB diagnostic and drug-resistance detection tests, according to country NTP guidelines

Assay name	Cambodia	China	Lao PDR	Mongolia	Philippines	PNG	Viet Nam
Xpert Ultra	Everyone with presumptive TB	Everyone with presumptive TB	Everyone with presumptive TB	Everyone with presumptive TB	Everyone with presumptive TB	Everyone with presumptive TB	High risk groups with presumptive TB or abnormal CXR
Truenat MTB Plus	Everyone with presumptive TB	NA	NA	NA	Everyone with presumptive TB	Adults with presumptive TB	Everyone with presumptive TB
TB-LAMP	NA	Everyone with presumptive TB	NA	NA	Everyone with presumptive TB	NA	NA
LF-LAM	NA	NA	NA	NA	All PLHIV	NA	All PLHIV with presumptive TB; hospitalised or severely ill PLHIV
Smear microscopy	Everyone with presumptive TB in areas without NAAT coverage	Everyone with presumptive TB in areas without NAAT coverage	NA	Everyone with presumptive TB in areas without NAAT coverage	Everyone with presumptive TB in areas without NAAT coverage	Everyone with presumptive TB in areas without NAAT coverage	Everyone with presumptive TB in areas without NAAT coverage
LPA 1st line	NA	Smear- or culture-positive individuals with unknown RIF-resistance	NA	People with poor treatment response; people with previous TB	Everyone with confirmed RR-TB	Everyone with confirmed RR-TB; people with poor treatment response	Everyone with confirmed RR-TB; people with poor treatment response
LPA 2nd line	NA	NA	Everyone with confirmed TB irrespective of RIF-resistance	Everyone with confirmed RR-TB; people with poor treatment response; people with previous TB	Everyone with confirmed RR-TB	Everyone with confirmed RR-TB; people with poor treatment response	Everyone with confirmed RR-TB; people with poor treatment response
LPA PZA	NA	NA	NA	NA	NA	Everyone with confirmed RR-TB; people with poor treatment response	NA
Xpert MTB/XDR	Everyone with confirmed RR-TB; people deemed at risk of drug resistance by clinician	NA	Everyone with confirmed RR-TB	Everyone with confirmed RR-TB; people with poor treatment response; people with previous TB	Everyone with confirmed RR-TB; people with poor treatment response	Everyone with confirmed RR-TB; people with poor treatment response; smear-positive individuals with suspected drug resistance	Everyone with confirmed RR-TB; people with poor treatment response
MGIT liquid phenotypic drug susceptibility testing	Everyone with confirmed TB irrespective of RIF-resistance	Everyone with confirmed RR-TB	NA	Everyone with confirmed TB irrespective of RIF-resistance; people with poor treatment response; people with history of TB	Everyone with confirmed RR-TB	Everyone with confirmed RR-TB	Everyone with confirmed RR-TB; people with poor treatment response; everyone with TB relapse; people prescribed BPaL regimen
Löwenstein–Jensen medium solid drug susceptibility testing	Everyone with confirmed TB irrespective of RIF-resistance	Everyone with confirmed TB irrespective of RIF-resistance	Everyone with confirmed TB irrespective of RIF-resistance	NA	Everyone with confirmed RR-TB	Everyone with confirmed RR-TB	NA
Middlebrook medium solid drug susceptibility testing	NA	NA	NA	NA	NA	Everyone with confirmed RR-TB	NA
Whole genome sequencing	NA	NA	NA	People with discordant NAAT and culture results	NA	NA	NA
Targeted next generation sequencing	NA	NA	NA	NA	NA	NA	People with poor treatment response; people with discordant NAAT and culture results
Other assay for genomic drug susceptibility testing	NA	Everyone with confirmed TB irrespective of RIF-resistance	NA	NA	NA	NA	NA

*CXR* chest X-ray, *LF-LAM* lateral flow lipoarabinomannan, *NA* test not available, *NAAT* nucleic acid amplification test, *PLHIV* people living with HIV, *PNG* Papua New Guinea, *RR-TB* rifampicin-resistance tuberculosis, *TB* tuberculosis

Diagnostic tests without capacity for drug-resistance testing (e.g., TB-LAMP [Eiken Chemical Company Ltd, Japan]) are not widely used in the region. Recommended as one option to replace smear microscopy [18], TB-LAMP is only programmatically implemented in China and the Philippines. Lateral-flow urine lipoarabinomannan (LF-LAM) testing is available only in the Philippines and Viet Nam (Table 1); this may be because the test is seen as lower priority due to the region’s relatively low HIV-TB burden. Smear microscopy continues to be used for TB diagnosis and treatment monitoring by all seven countries, except Lao PDR, which only uses smear microscopy among Xpert-positive cases for treatment monitoring. In interviews, many country representatives explained that smear microscopy is reportedly used for initial diagnosis in areas without access to NAAT testing, typically in countries’ more rural and remote locations.

Regarding add-on drug-resistance detection diagnostic tests, first-line line probe assays (LPA) are available widely across the region, although deployed in specific patient populations (Table 1). LPA’s intended-use population varies between countries and includes everyone with confirmed rifampicin-resistant TB (RR-TB), people exhibiting a poor treatment response, people with previous TB (Mongolia only), and in China people who were initially diagnosed via smear microscopy and therefore have no drug-resistance information available (Table 2). All countries using second-line LPA recommend it among people with confirmed RR-TB, except Lao PDR where everyone with confirmed TB is eligible to receive it. LPA PZA is only recommended for use in Papua New Guinea (Table 1) but no LPAs have been used in this country since 2020 due to technical and infrastructure problems. Use of Xpert MTB/XDR is expanding in the region, with all countries except China reporting some level of implementation. In these six countries, everyone with confirmed RR-TB is eligible for this test, as well as those exhibiting a poor treatment response (Table 2). Some countries mentioned increasing concerns regarding isoniazid resistance as a motivator for physicians to order this test, even among TB patients who are not mandated to undergo Xpert MTB/XDR testing by the NTP.

### Access to WHO-recommended rapid diagnostic tests

Access to WHO-recommended rapid diagnostic (WRDs) tests with drug-resistance detection capabilities (i.e. WHO-recommended NAATs that detect TB and drug-resistance mutations) is a key element of the Regional Framework for ending TB [14]. To increase patient access to WRDs, the framework calls for their decentralisation in TB programmes. This could take the form of placing tests outside of centralised reference laboratories and relocating them to peripheral sites, or enacting efficient sample transport systems [14]. It is critical that people with presumptive TB have easy access to these highly accurate WRDs to allow timely initiation of effective treatment.

Table 3 displays the levels of the health care system at which WRDs are available in each of the seven key countries. Running WRDs in peripheral settings is not common throughout the Region. In Mongolia, Papua New Guinea, and Viet Nam, WRDs are available at peripheral levels in some areas, but not throughout the countries, whereas in the Philippines, WRDs are available at peripheral level in all regions. In China, WRDs are available in designated TB hospitals, which are located at the district level; WRD and CFDA-approved NAAT coverage is good in the eastern regions of the country, but less so in the west of the country. In Lao PDR, samples for testing are collected at peripheral sites and are then transported to district-level laboratories where GeneXpert modules are situated. In Cambodia, WRDs are available at most district-level sites, with the NTP aiming to have coverage throughout the country. WRD placement and access can be further contextualised using WHO’s data on TB Diagnostics Benchmarks [19]. Table 4 shows country responses to some key indicators, although overall, reporting on the benchmarks is still uneven across the Region.

**Table 3:** Access to WHO-recommended rapid diagnostic tests with drug-resistance detection as initial TB diagnostic test

Level—region	Cambodia	China	Lao PDR	Mongolia	Philippines	PNG	Viet Nam
Peripheral level—some regions				X		X	X
Peripheral level—throughout country					X		
Intermediate laboratories or district hospitals—some regions	X	X	X				
Intermediate laboratories or district hospitals—throughout country				X			X
Central laboratory or reference hospital—some regions				X			
Central laboratory or reference hospital—throughout country	X		X				X

**Table 4:** Benchmarks 3, 4, 5, and 9 of the WHO standard for diagnostics

Benchmark	Cambodia	China	Lao PDR	Mongolia	Philippines	PNG	Viet Nam
3. Percentage of districts in which all facilities have a TB diagnostic algorithm requiring use of a WRD test as initial diagnostic test for all people with presumptive TB	NR	NR	NR	97%	100%	NR	26%
4. Percentage of primary healthcare facilities with access to WHO-recommended rapid diagnostic tests	NR	NR	100%	0.37%	100%	NR	NR
5. Percentage of new and relapse cases tested using a WRD test as the initial diagnostic test	NR	74%	NR	90%	NR	NR	50%
9A. Percentage of people with bacteriologically confirmed PTB with test results for rifampicin	NR	89%	100%	96%	83%	NR	90%
9B. Percentage of people with rifampicin-resistant TB and with available test results for susceptibility to fluoroquinolones	100%	34%	93%	82%	12%	100%	84%
9C. Percentage of people with rifampicin- and fluoroquinolone-resistant TB and with available test results for susceptibility to bedaquiline	NR	5.3%	NA*	88%	NR	NR	54%
9D. Percentage of people with rifampicin- and fluoroquinolone-resistant TB and with available test results for susceptibility to linezolid	NR	13%	NA^	88%	NR	NR	52%

Data extracted from [19]

*NA* not applicable, *NR* not reported, *PNG* Papua New Guinea, *PTB* pulmonary tuberculosis, *WRD* World Health Organization-recommended rapid diagnostics

*Zero reported cases of people with rifampicin- and fluoroquinolone-resistant TB

^ Zero reported cases of people with rifampicin- and fluoroquinolone-resistant TB

### Drug susceptibility testing usage and in-country availability

Phenotypic drug susceptibility testing (pDST) remains a critical tool across the region and is programmatically implemented among the seven countries. All countries except Lao PDR reported using mycobacterial growth indicator tube ([MGIT], BD, USA) liquid culture for pDST, and all countries except Mongolia and Viet Nam also routinely use Löwenstein–Jensen (LJ) solid culture for pDST. In Lao PDR, the national reference laboratory is using a customised LJ solid culture system called M-KIT. Across the Region, pDST may be used in a variety of eligible populations (Table 2), but use is often elective according to NTP guidelines, as individuals would have already received a NAAT with some DST capacity. However, in Lao PDR, Papua New Guinea, and Viet Nam, their respective pDST tests are mandatory in eligible populations.

Sequencing-based genotypic DST (gDST), such as targeted next generation sequencing (tNGS), is not yet widely implemented in the Region. China is using it for research and surveillance purposes, but not for DST (Table 2). In Viet Nam, tNGS is being rolled out (Table 1), with the priority population being people with a poor treatment response, followed by people with discordant NAAT and culture results (Table 2); the eventual goal there is for everyone with RR-TB to undergo tNGS. Mongolia is currently introducing whole genome sequencing for drug susceptibility testing (DST).

The drugs for which susceptibility can be tested in each country are reported in Table 5. In general, DST capacity is available for first-line TB drugs, i.e. for treating drug-susceptible TB. All countries can perform DST for isoniazid, rifampicin, and ethambutol, three components of the standard 6-month drug-susceptible TB treatment regimen, and six countries have some capacity to query pyrazinamide susceptibility, the fourth component [20]. All countries also have DST for fluoroquinolones and amikacin/streptomycin. However, for new and re-purposed drugs like bedaquiline, delamanid, or linezolid, some countries have neither pDST nor gDST available. Notably, in China, commercial DST assays for bedaquiline are not yet available; as a result, some laboratories have established in-house minimum inhibitory concentration assays. However, quality assurance (QA) is lacking, with low reliability of these assays as a result. In multiple interviews, countries’ NTP laboratory staff mentioned the costs of pDST and gDST platforms and test reagents as limiting factors for DST uptake. This is true even as most countries are receiving Global Fund support for DST, aside from China (domestic funding) and Mongolia (some support from the Japanese government). While all countries’ NTPs have established eligibility criteria for pDST and gDST, it was widely acknowledged that these criteria are not always implemented in practice.

**Table 5:**
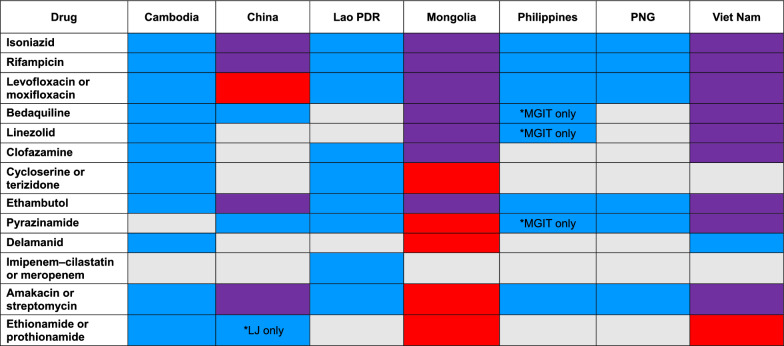
TB drugs for which drug susceptibility testing is available in seven countries (phenotypic and genotypic)

*Blue* susceptibility testing using phenotypic DST only. *Red* susceptibility testing using genotypic DST only. *Purple* susceptibility testing available on both phenotypic and genotypic DST. *Grey* susceptibility testing is not available

*DST* drug susceptibility testing, *LJ* Löwenstein–Jensen medium, *MGIT* Mycobacteria growth indicator tube, *PNG* Papua New Guinea

Data in Table 4 also include WHO Diagnostics Benchmarks [19] related to drug susceptibility testing. It shows that most bacteriologically confirmed TB patients in the region have access to gDST for rifampicin resistance; however, among rifampicin-resistant TB cases, the proportion tested for fluoroquinolone resistance varies substantially. Only Cambodia has reported universal access among these patients for the detection of fluoroquinolone resistance. In China, the NTP’s goal is that within three years, all confirmed patients with rifampicin-resistant TB should undergo testing for fluoroquinolone resistance, though at present this is not yet achieved. Among TB patients with pre-XDR TB, the proportion receiving testing for bedaquiline or linezolid resistance also varies, or data are not available, except for in Mongolia where whole genome sequencing is being used for DST.

### Laboratory networks

Tuberculosis testing laboratory networks among the seven countries are generally structured similarly. There is a central, national reference laboratory at the highest level, which has overall authority over intermediate- and peripheral-level laboratories, with QA and quality management activities being among their key responsibilities. Most countries’ laboratory networks operate over three levels, while China and Viet Nam have an additional fourth level between the intermediate and central levels. All seven countries reported that their national reference laboratories oversee QA activities throughout the country (Supplementary Table 1), and most formally collaborate with a WHO TB Supranational Reference Laboratory for proficiency panel testing, introducing new diagnostic tests, and consultation on challenging cases requiring additional testing. See Supplementary Results for additional details. Hiring and retaining qualified laboratory personnel was not cited as a major challenge in the Region, although staff workload may be an issue (see Supporting Results, Supplementary Table 2).

If a healthcare facility does not have capacity to perform the initial diagnostic test on the premises, the sample must be transported to a TB testing laboratory. Samples are transported using a variety of methods detailed in Supplementary Table 3. Across the region, people providing sputum for TB testing produce them on-site and under direct supervision from healthcare staff. Specimen transport approaches vary across countries, with some countries using TB-specific courier networks, while others have more integrated approaches. Details can be found in Supplementary Results.

## Laboratory information systems and test result reporting tracking

Information systems across the region vary between the seven countries, but no country is using an end-to-end fully electronic system. Specimen tracking logbooks are site-specific and are paper-based, although some countries complement this with bespoke Excel workbooks (Table 6). Test requisition forms are generally paper-based, although some countries have TB-specific portals for tracking test orders. When laboratories return test results to the ordering physician, a variety of practices are observed: in some countries, test results may be informally notified to the treating physician via SMS or email, with the physical completed test requisition form to follow, whereas in China, Mongolia, and the Philippines, electronic reporting systems are used. Previously, GX Alert was used for delivering results tested on the GeneXpert platform, but none of the seven countries currently report using the system. Further details on information tracking, test requisition, results delivery, and data sharing with NTPs can be found in the Supplementary Results.

**Table 6:** TB data systems and information capture in seven countries

Activity	Cambodia	China	Lao PDR	Mongolia	Philippines	PNG	Viet Nam
Format of test requisitions received from healthcare workers and/or TB clinics at TB testing laboratories	Physical test requisition form	Electronic via laboratory information systems	Physical test requisition form	Physical test requisition form; electronic fit-for-purpose portal that can be accessed online	Physical test requisition form; electronic fit-for-purpose portal that can be accessed online	Physical test requisition form	Physical test requisition form
Method NTP uses for tracking requisitioned TB tests	Physical	Electronic via laboratory information systems	Physical	Electronic fit-for-purpose portal that can be accessed online	The NTP does not track ordered tests	The NTP does not track ordered tests	Electronic (custom Excel logbook)
Method TB test results are returned to healthcare workers	Physical	Electronic via hospital information systems	Electronic via messaging app; physical	Electronic via fit-for-purpose portal that can be accessed online; physical	Electronic via fit-for-purpose portal that can be accessed online or via email; physical	Physical	Electronic via messaging app or email; physical
Method NTP uses for tracking TB test results	Physical	Electronic via TB management information system	Physical	Electronic fit-for-purpose portal that can be accessed online	The NTP does not track tests results	Physical	Electronically (custom Excel logbook)

*NTP* national tuberculosis programmes, *PNG* Papua New Guinea, *TB* tuberculosis

## Conclusions

The burden of TB in these seven priority Western Pacific Region countries is huge, and meeting targets set out in the Western Pacific Regional Framework to End TB will be challenging. Countries have made substantial investments in their TB diagnostic and drug-resistance detection armamentaria, with WRDs now readily available for people with presumptive TB in much of the region. This investment is yielding actionable results, such as high coverage for rifampicin-resistance testing for most people with bacteriologically confirmed TB across the region. However, access to WRDs in remote areas is still limited in most countries, and the use of low-sensitivity smear microscopy to diagnose TB continues. This places many people with presumptive TB in remote settings at an inequitable disadvantage, as they may not be able to access highly accurate molecular tests with some drug-resistance detection capacity: this decreases their probability of receiving the most appropriate treatment course. Sample transportation systems, laboratory information systems, and workforce show variability across the region, emphasising the need for tailored approaches specific to each country.

Countries generally have good molecular and pDST options available for assaying resistance to key first-line TB drugs, but limited capacity to test new and re-purposed drugs; this means countries may not have an accurate understanding of local drug-resistance epidemiology. As 6-month all-oral drug-resistant TB treatment regimens [21] (i.e. BPaLM) are increasingly being rolled out, DST for these options must also become commercially available and adopted by national programmes. A transition of laboratory information systems to become fully electronic would likely facilitate record-keeping and may improve care [22, 23], but the region is still typically using a mix of paper-based and electronic systems. Some countries, e.g., China, Mongolia, and Viet Nam, are investing in case-based electronic information systems that can track samples from collection to test result. Once these systems are implemented, this will likely improve NTPs’ abilities to plan and prioritise placement and procurement of test kits, reagents, and personnel.

Having completed an initial assessment via the structured surveys and interviews, the next steps for the seven countries is to design action plans to act on these findings. However, the recent American executive branch’s dismantling of USAID and resultant upheavals to the global health funding landscape [24] may leave NTPs in the Region struggling to maintain their regular testing programs; this disruption to routine services has already been reported in the African Region [25]. The new reality will likely negatively impact NTPs’ ability to roll out newer assays and improved information systems. In the longer term, as countries are forced to find local sources of investment for TB services, this may ultimately result in their increased resilience and sustainability [26]. This is, however, likely of little consolation to the hundreds of thousands of individuals with presumptive TB who require TB and drug-resistance testing now and in the immediate future.

This work had strengths and limitations. Strengths include the inclusion of data from the seven countries with the largest TB burden in the Western Pacific Region. NTPs had multiple opportunities to contribute and correct data in this account. This provides an accurate and epidemiologically relevant snapshot of current TB testing and laboratory services. As well, the programmatic successes and challenges described above are likely shared by other countries in the Region. Regarding limitations, the findings are specific to the public sector NTP work only, and do not reflect diagnostic and laboratory services available only in the private sector or, generally, in research-use only settings. In some included countries, the private health sector contributes to TB case notifications, so this report may not indicate the complete extent of available services. Finally, the report’s findings are reflective of the time in which they were captured: impacts of recent major global health funding reductions are not reflected herein.

This evaluation highlights successes and persistent gaps to advocate for continued investment in TB services regionally. Ultimately, the findings from this assessment and the workshop will be synthesised to generate recommendations and country action plans to improve integrated diagnostic and laboratory services in seven priority Western Pacific Region countries.

## Supplementary Information

Additional file 1.

## Data Availability

No datasets were generated or analysed during the current study.

## Abbreviations

CFDA: Chinese Food and Drug Administration
COVID-19: Coronavirus disease 2019
Laos PDR: Lao People’s Democratic Republic
LF-LAM: Lateral flow lipoarabinomannan
LJ: Löwenstein–Jensen medium
LPA: Line probe assay
MDR-TB: Multidrug-resistant tuberculosis
MGIT: Mycobacterial growth indicator tube
NA: Not applicable
NAAT: Nucleic acid amplification test
NTP: National tuberculosis programme
pDST: Phenotypic drug susceptibility testing
PNG: Papua New Guinea
QA: Quality assurance
RR-TB: Rifampicin-resistant tuberculosis
RIF: Rifampicin
TB: Tuberculosis
tNGS: Targeted next generation sequencing
Truenat: Truenat MTB Plus
WGS: Whole genome sequencing
WHO: World Health Organization
WPRO: Western Pacific Regional Office
Xpert: Xpert MTB/RIF or Xpert MTB/RIF Ultra
