# Supplementary material for: Progress in tuberculosis diagnosis and laboratory services in the Western Pacific Region: a situational analysis of seven high tuberculosis burden countries
Source: Trop Med Health. 2026 May 18;54:86. doi: 10.1186/s41182-025-00898-z (PMC13182110; doi:10.1186/s41182-025-00898-z)
Supplement: Supplementary file 1 — Additional file 1. [file 41182_2025_898_MOESM1_ESM.docx]

*Supplement – Tropical Medicine and Health*

**Progress in tuberculosis diagnosis and laboratory services in the Western Pacific Region: a situational analysis of seven high tuberculosis burden countries**

Emily Lai-Ho MacLean^1,2^, Kyung Hyun Oh^3^*, Kalpeshsinh Rahevar^3^, Carl-Michael Nathanson^4^, Alexei Korobitsyn^4^, Satoshi Mitarai^5^, Seiya Kato^5^, Fukushi Morishita^3^, Huong Thi Gian Tran^3^, Rajendra Prasad Hubraj Yadav^3^, for the Tuberculosis Diagnosis and Laboratory Services Western Pacific Regional Assessment Consortium.

^1^NHMRC Clinical Trials Centre, Faculty of Medicine and Health, University of Sydney, Sydney, Australia.

^2^WHO Collaborating Centre for Tuberculosis, Sydney Infectious Diseases Institute, University of Sydney, Sydney, Australia.

^3^Western Pacific Regional Office, World Health Organization, Manilla, The Philippines.

^4^Global Tuberculosis Programme, World Health Organization, Geneva, Switzerland.

^5^The Research Institute of Tuberculosis, Japan Anti-Tuberculosis Association, Tokyo, Japan.

*Corresponding author

Regional Office for the Western Pacific, World Health Organization, Manila, Philippines.

Tel: +63 2 5289708; [ohk@who.int](mailto:ohk@who.int)

**Members of the Tuberculosis Diagnosis and Laboratory Services Western Pacific Regional Assessment Consortium**

| **Name** | **Affiliation** | **Country** |
| --- | --- | --- |
| Pheng Sok Heng | National Center for TB and Leprosy Control (CENAT) | Cambodia |
| Narith Ratha | Reference Laboratory National Center for TB and Leprosy Control (CENAT) | Cambodia |
| Serongkea Deng | Office of the WHO Representative in Cambodia | Cambodia |
| Xia Hui | National TB Reference Laboratory National Center for TB Prevention and Control  China Centres for Disease Control | China |
| Ou Xichao | National Center for TB Prevention and Control  China Centres for Disease Control | China |
| Pang Yu | Beijing Chest Hospital | China |
| Chen Zhongdan | Office of the WHO Representative in China | China |
| Boualay Norchaleun | National Center for Laboratory and Epidemiology Department of Communicable Disease Control Ministry of Health | Lao Peoples Democratic Republic |
| Souvimone Siphanthong | National Tuberculosis Control Center Department of Communicable Disease Control Ministry of Health | Lao Peoples Democratic Republic |
| Ratsamy Vongkhamsao | Department of Communicable Disease Control Ministry of Health | Lao Peoples Democratic Republic |
| Vilath Seevisay | Office of the WHO Representative in Lao Peoples Democratic Republic | Lao Peoples Democratic Republic |
| Sarantuya Ganbaatar | Surveillance and Prevention of HIV/AIDS and Tuberculosis Department of Public Health Ministry of Health | Mongolia |
| Uyanga Erdenebileg | Department of Tuberculosis Surveillance and Research National Center for Communicable Diseases Ministry of Health | Mongolia |
| Tsetsegtuya Borolzai | National TB Reference Laboratory National Center for Communicable Diseases Ministry of Health | Mongolia |
| Anuzaya Purevdagva | Office of the WHO Representative in Mongolia | Mongolia |
| Janet Gare | Surveillance and Outbreak - PNGIMR  National Department of Health | Papua New Guinea |
| Jennifer Dume | Central Public Health Laboratory  National Department of Health | Papua New Guinea |
| Jennifer Banamu | National Reference Laboratory  Port Moresby General Hospital | Papua New Guinea |
| Challa Negeri Ruda | Office of the WHO Representative in Papua New Guinea | Papua New Guinea |
| Ailene C. Espiritu | Disease Prevention and Control Bureau Department of Health | Philippines |
| Ramon Basilio | National TB Reference Laboratory  Research Institute of Tropical Medicine | Philippines |
| Dinh Van Luong | National TB Program National Lung Hospital | Philippines |
| Thomas Dale Hiatt | Office of the WHO Representative in the Philippines | Philippines |
| Clarissa Blanca Halum | Office of the WHO Representative in the Philippines | Philippines |
| Dinh Thi Huong | National TB Reference Laboratory Department of Microbiology National Lung Hospital | Viet Nam |
| Le Van Sang | General Department of Preventive Medicine  Ministry of Health | Viet Nam |
| Vu Quang Hieu | Office of the WHO Representative in Viet Nam | Viet Nam |

**Supplementary Results**

*Multiplex disease testing using GeneXpert*

The GeneXpert platform is used by all seven countries to run Xpert MTB/RIF Ultra. However, other cartridge-based assays are commercially available to detect additional diseases and conditions. Six of seven countries are using the GeneXpert platform to test for other conditions, in addition to TB. Lao PDR, Mongolia, the Philippines, Papua New Guinea, and Viet Nam have all used GeneXpert to test for COVID-19. China and Mongolia are using it to test for hepatitis C, while Lao PDR is using it to test for influenza. HIV viral load tests are available in China, Lao PDR, Mongolia, the Philippines, and Papua New Guinea. The platform is also being used to test for chlamydia, gonorrhoea, *Staphylococcus aureus*, and MRSA in China; for HPV in the Philippines; and early infant detection of HIV in Papua New Guinea.

*Role of national reference laboratories*

All seven countries reported that their NRL (i) supervises intermediate-level laboratories' implementation and use of bacteriological methods and the laboratories' performance monitoring of peripheral laboratories; (ii) undertakes QA of all procedures performed at intermediate-level laboratories including microscopy, WRDs, culture, and DST; and (iii) ensures that appropriate human resources development programmes are in place, including training, retraining, and competency assessment. All countries except Lao PDR reported that their NRLs have a formal collaboration with a WHO TB Supranational Reference Laboratory for proficiency panel testing; support in implementing and validating new diagnostics; assistance with laboratory development and expansion strategies; and referral for challenging cases that require specialized testing. Additionally, except in Lao PDR and Papua New Guinea, all NRLs undertake operational and applied research pertaining to the laboratory network, and coordinate this with the requirements and needs of the NTP. Countries indicated that intermediate and peripheral laboratories also participated in QA activities; details are provided in Table S1.

*Laboratory personnel*

All seven countries reported that most new positions are full-time, and all but one reported low turnover (Table 10). Papua New Guinea experience high staff turn-over, particularly in laboratories that are not fully government funded, e.g., laboratories supported by faith-based organisations. Staff workload was indicated as an issue in some countries. In China, the interviewee noted that staff regularly work overtime to ensure all TB-related duties are completed. In Viet Nam, smaller district laboratories often rely on a single person conducting testing, causing operations to come to a halt in the event of their absences.

*Sample transport*

If a healthcare facility does not have capacity to perform the initial diagnostic test on the premises, the sample must be transported to a TB testing laboratory. Samples are transported using a variety of methods detailed in Table 8. In all seven countries, people undergoing testing provide sputum samples on-site after receiving instruction or under direct supervision from clinic or hospital staff. In every country except Viet Nam, samples collected at home may also be brought to site (Table 8). In all countries, facility staff transport samples to TB testing laboratories, while some also hire couriers for this purpose. Papua New Guinea is conducting a Diagnostic Network Optimisation in collaboration with FHI to strengthen their sample transportation network and pilot various approaches. Mongolia and Viet Nam have transportation systems specific to TB. The Philippines has established a specimen transport system where motorbike riders, so-called STRiders (specimen transport riders), transport clinical specimens from collection sites to laboratories; the system also transports specimens for HIV and COVID-19 testing and is implemented throughout the country. Cambodia has a generic transportation system and in China there are regional variations.

*Information systems*

Sites in all countries maintain physical sample logbooks for tracking purposes. In Lao PDR, messaging apps are also used to aid in sample transportation, particularly to alert testing laboratories that samples are being sent from district centres. Mongolia is rolling out an electronic system called ‘2-B’ which can monitor transportation in real-time (Box 2). The Philippines, Papua New Guinea, and Viet Nam use bespoke Excel logbooks to document information regarding samples that have been sent for further testing.

In all countries except China, TB testing laboratories rely to varying degrees on paper-based test requisition forms (Table 6). Often it is clinic staff who are responsible for transporting samples and requisition forms to the designated testing laboratory. In Cambodia, Lao PDR, Mongolia, and Papua New Guinea, patients may have to deliver their samples and forms. Cambodia, the Philippines, and Viet Nam also use contracted courier companies.

In Mongolia, laboratories are transitioning to ‘2-B’, but many are still reliant on paper-based systems for certain test orders, e.g., smear microscopy. In China, there is no single standard portal used by all laboratories; rather, TB laboratories all use their own unique information systems, which are linked to the hospitals with which they are affiliated.

NTPs in Mongolia, China, and Viet Nam can track TB tests that have been requested through electronic means, whereas in Cambodia and Lao PDR, NTP staff or couriers collect paper records of tests ordered at lower levels of healthcare system and report this data to higher levels. In the Philippines and Papua New Guinea the NTPs do not track ordered tests.

Test results are returned to the requesting provider using various methods (Table 6). Cambodia uses a paper-based system for reporting results. In Lao PDR and Viet Nam, healthcare workers typically receive an informal electronic notification via email or messaging app with the result, with a formal paper-based report form delivered subsequently. In China, Mongolia, and the Philippines, electronic reporting systems are used, and in Papua New Guinea, GX Alert was previously used but due to funding constraints the system is not currently operating, with paper-based reporting used instead. Viet Nam also used GX Alert previously, but due to functionality issues no longer use it; the NTP is currently developing its own TB laboratory information management system.

In Cambodia, Lao PDR, and Papua New Guinea, sites summarise test results and submit to province level laboratories, who collate results to the NRL; in Papua New Guinea, certain sites report directly to the NRL. In Lao PDR, NTP staff may travel to sites to collect test result data from physical logbooks, particularly for smear microscopy. In Cambodia, clinic staff may deliver results themselves to the NTP. According to our survey results, the Philippines’ NTP does not track TB test results. The remaining country NTPs can access TB test results electronically through information systems.

*Electronic information system in Mongolia*

The NTP in Mongolia is rolling out an electronic, cloud-based information system called ‘2-B’. The system allows monitoring of patient registration, test orders, test results, prescribed regimens, drug management, contact screening, and TB preventive therapy administration in near real-time. The system will eventually become integrated with other health systems, such as eHealth (hospital system) throughout the country.

**Table S1:** Quality assurance and quality management duties undertaken by TB testing laboratories in peripheral and intermediate settings among seven countries. EQA – external quality assurance; QA – quality assurance; QC – quality control; PNG – Papua New Guinea; WRD – World Health Organization-recommended rapid diagnostics.

| **Level – region** | **Cambodia** | **China** | **Lao PDR** | **Mongolia** | **Philippines** | **PNG** | **Viet Nam** |
| --- | --- | --- | --- | --- | --- | --- | --- |
| **Peripheral level – following appropriate QC and QA procedures** | X | X |  | X | X |  | X |
| **Peripheral level – participating in EQA programmes (e.g. blinded rechecking, panel testing, and supervisory visits)** | X | X | X | X | X | X | X |
| **Intermediate laboratories – train lab technicians and supervise peripheral-level staff in microscopy and WRDs** | X | X | X |  | X | X | X |
| **Intermediate laboratories – engage in proficiency testing and quality improvement activities for peripheral laboratories** | X | X |  |  | X |  |  |
| **Intermediate laboratories – other** |  |  |  | X |  |  |  |

**Table S2:** Status of personnel in laboratories performing TB testing in seven countries. QC – quality control; PNG – Papua New Guinea; TB – tuberculosis.

| **Criterion regarding laboratory personnel in TB laboratories** | **Cambodia** | **China** | **Lao PDR** | **Mongolia** | **Philippines** | **PNG** | **Viet Nam** |
| --- | --- | --- | --- | --- | --- | --- | --- |
| Type of contracts for new staff | Full-time | Full-time | Full-time | Full-time | Full-time | Full-time | Full-time |
| Staff turnover rate | Low | Low | Low | Low | Moderate | High | Low |
| Sufficient to complete all TB tests ordered in a day | Yes | Partially | Yes | Yes | Yes | No | Partially |
| Sufficient to complete all TB-related reporting and data entry | Yes | Partially | Yes | Yes | Partially | No | Partially |
| Sufficient to complete all maintenance, calibration, QC activities | Yes | Partially | Yes | Yes | Yes | No | Partially |

**Table S3:** Sample transport methods in seven countries. X indicates the method is being used. PNG – Papua New Guinea.

| **How are samples transported if a sample cannot be tested on-site?** | **Cambodia** | **China** | **Lao PDR** | **Mongolia** | **Philippines** | **PNG** | **Viet Nam** |
| --- | --- | --- | --- | --- | --- | --- | --- |
| **Site contracts couriers to transport samples** | X |  |  |  | X |  | X |
| **Site staff transport samples** | X | X | X | X | X | X | X |
| **Patients transport their own samples** | X |  | X | X |  | X |  |
| **Testing laboratories collect samples from sites** |  | X | X |  |  |  |  |
| **Other method (specify)** |  |  |  |  | Specimen transport riders (Striders) who are motorcycle drivers engaged  thru Global Fund. | DNO (diagnostic network optimisation) |  |
